# How tensions between parents’ values influence decisions about their children’s nutrition: a qualitative study in disadvantaged neighbourhoods

**DOI:** 10.1186/s12939-025-02712-y

**Published:** 2025-12-05

**Authors:** Noa van den Brink, Valentijn T. Visch, Nicolien D. M. Dinklo, Ashley J. P. Smit, Heleen Bouma, Marina Bos-de Vos

**Affiliations:** 1 Faculty of Industrial Design Engineering, Delft University of Technology, Delft, The Netherlands; 2 Department of Public Health, Erasmus Medical Centre, Rotterdam, The Netherlands; 3 Department of Neonatal and Pediatric Intensive Care, Erasmus Medical Centre - Sophia Children’s Hospital, Rotterdam, The Netherlands; 4 Muzus, Delft, The Netherlands

**Keywords:** Healthy nutrition, Nutrition behaviour, Value tensions, Disadvantaged neighbourhoods, Stress, Constraints, Protective factors, Children, Social support

## Abstract

**Background:**

Parents feel a responsibility to provide a healthy start for their young children, but struggle to realise this. Especially in disadvantaged neighbourhoods, parents face challenges such as financial strain, stress, and isolation. Facing these challenges can contribute to tensions between parents’ values, such as balancing family harmony with healthy food choices. Such value tensions may negatively affect the nutrition decisions parents make for their children. Nutrition interventions often fail to address these value tensions, which contributes to their relatively low uptake and impact. By understanding the tensions parents face between their values, the present research offers recommendations for future nutrition interventions to encourage healthy nutrition decisions in disadvantaged situations.

**Methods:**

We conducted a qualitative interview study using semi-structured interviews with 20 parents of children aged zero to four years, living in disadvantaged neighbourhoods. Transcripts were analysed inductively to identify value tensions, *stressors* that trigger them, and *protective factors* that mitigate their impact.

**Results:**

Six key value tensions parents experienced regarding nutrition were identified, which emerged in situations involving, for example, stress, low income, or limited social support. The three most common tensions included balancing the value of the dietary health of the child with the values of *enjoyment of the child*,* convenience for the parent*, and *well-being of the parent*. Our analyses showed that the value tensions were triggered by specific stressors, such as challenging child behaviour, unhealthy food provided by friends or family, and lack of me-time for the parent. The participants reported relief from stressors and the resulting value tensions by relying on *protective factors* such as social and material support, including informal household support and access to healthy, convenient foods.

**Conclusions:**

This study provides insights into how *value tensions*,* stressors*,* and protective factors* influence parents’ nutrition decisions for their children. By addressing these value tensions, alongside other influences such as structural barriers, nutrition interventions may become more fitting and motivating for parents within their specific contexts. In addition to general recommendations on value-based intervention design, our findings offer specific guidance for developing tailored nutrition interventions for families in disadvantaged neighbourhoods.

**Supplementary Information:**

The online version contains supplementary material available at 10.1186/s12939-025-02712-y.

## Background

A healthy diet is essential for supporting a child’s cognitive, physical, and emotional growth [1]. Additionally, a diet rich in fruits and vegetables and low in sugary foods is crucial in the early years of life, as it helps reduce the risk of developing chronic diseases like cardiovascular conditions [2]. Parents generally feel a responsibility to provide their child with a healthy start in life [3]. However, maintaining a healthy diet can be challenging for parents. Especially, families in disadvantaged neighbourhoods face challenges in managing a healthy lifestyle. Parents may be limited in supporting their child’s dietary health due to challenges in their personal and neighbourhood context, including financial constraints, limited time for shopping and cooking, low social support, stress due to safety concerns, and poor transportation access to food stores [4–8]. Therefore, it is crucial to support these families with young children from disadvantaged neighbourhoods in improving their children’s health outcomes now and for their future.

Many interventions have been designed to support a healthy dietary intake in young children [9–11]. However, most current interventions mainly focus on providing knowledge about healthy nutrition [12], without offering opportunities to apply that knowledge in the daily life context of the parents and family life. Interventions typically ignore that parents’ nutrition behaviour is not only influenced by valuing healthy nutrition but also by other, potentially competing, values. Here, values are principles of importance to parents that guide parents’ behaviour and resulting nutrition decisions [13]. Addressing one value can lead to actions that contend with another value, and as challenges, like a child’s behaviour, bring these values into juxtaposition, a tension arises [14]. Thus, a value tension occurs when values compete, making it difficult to satisfy both simultaneously. For example, when children have a limited range of accepted foods, parents may prioritise their children’s taste preferences over healthier options. This reflects a tension between values like ‘family harmony’ and ‘health’ [15]. This value tension manifests through negative, distressing emotions in parents [16, 17], where providing less healthy foods to protect family harmony can lead to feelings of guilt or anxiety, as health is still valued. Moreover, the challenges increasingly present in disadvantaged neighbourhoods (e.g. poverty, inadequate housing, and safety concerns [4, 6, 18]) further affect parents’ actions driven by underlying value tensions. For instance, stress, such as due to financial issues, has been shown to promote unhealthy nutrition practices [19].

Involving parents’ values and context in interventions can enhance their relevance and increase parents’ motivation to engage, as these interventions then better align with the parents’ needs [20–23]. To design effective interventions that address the challenges parents encounter when values are in tension, it is essential to understand the specific conditions that give rise to or relieve these tensions. The context in which values appear is critical for understanding their meaning, as it influences how values are formed and expressed [24, 25]. Contextualising values also ensures they are not too abstract for intervention design [26]. While considerations such as convenience, affordability, and children’s preferences are well documented as shaping parents’ nutrition decisions [17, 27], a better understanding is needed of how parents are challenged or supported in acting according to their values in nutrition decisions, so that interventions can address these influences. Additionally, parents may encounter factors that reduce challenges and related value tensions, such as social support, or develop contextually beneficial responses to these value tensions [4, 28]. Understanding these factors in the parents’ context is especially important for identifying what can support parents in their nutrition decisions, rather than only focusing on what is lacking. This shifts the focus from individual deficits to strengths and available resources that can result in more equitable interventions [29].

To inform interventions targeting children’s diet, this research aims to understand value tensions related to nutrition decisions experienced by parents of children aged zero to four years, residing in disadvantaged neighbourhoods. It also investigates how parents are challenged or supported in acting on their values regarding their child’s healthy nutrition, such as through stress and social support.

## Methods

### Study design

This study employed a qualitative, exploratory design to investigate how parents experience value tensions related to nutrition. We define values not just as abstract principles or motivational goals, but as *lived experiences* that are expressed, shaped, and experienced in everyday life situations and behaviours [24, 25]. This framing is particularly suited to explore how value tensions around nutrition are shaped in everyday family contexts, where stress and other influences affect decision-making. These subjective and context-dependent experiences are best examined through qualitative methods, which can capture depth and nuance. Therefore, we conducted semi-structured interviews. The interviews were preceded by *sensitisation exercises* [30] that prepared participants for the interviews by creating awareness of their experiences of nutrition choices. These exercises were sent to participants via WhatsApp over four days, asking about their experiences, needs, and values regarding nutrition for their family (Additional file 1: Table S1). These exercises served as prompts to guide interview questions rather than as direct data collection tools, helping participants reflect in advance and enter the conversation with a deeper understanding of their experiences.

Informal conversations with parents during the recruitment phase helped us identify common language and sensitivities around nutrition. Insights from these exchanges informed the development of interview questions and sensitisation materials, ensuring they resonated with how parents naturally discuss nutrition and related topics.

The sensitisation exercises and interview questions were also pilot-tested with a parent to assess their clarity and relevance.

### Recruitment and participants

Participants were recruited based on the criteria of living in a selected disadvantaged neighbourhood in Rotterdam, the Netherlands, and having at least one child aged zero to four years old. This age range was chosen to focus on the earliest years of life, during which healthy nutrition is vital for the child’s development and disease prevention [1, 2]. The neighbourhood was selected based on data from Dutch government agencies CBS and RIVM, which identified its higher vulnerability compared to the national average, characterised by unhealthy lifestyles, elevated stress, and lower socioeconomic status as a composite measure of income, education, and occupation [31, 32]. The selected neighbourhood had 63,670 residents at the time of writing the paper. Among households, 35% included children, 59% had a migration background, and 13% lived at the social minimum [33]. To avoid excluding individuals experiencing disadvantage not captured by individual socioeconomic status alone, such as highly educated single parents with limited support, we opted for neighbourhood-based recruitment. While this approach may risk reinforcing negative perceptions of specific areas, it avoids the implication and related stigma that individual characteristics cause poor health outcomes [34, 35]. Participants had to be at least 20 years old to exclude teenage parents, who may face additional challenges that warrant separate investigation. Recruitment methods included engaging potential participants through established relationships with key community figures, visiting activities at community centres, conducting open conversations about health in public places like playgrounds and malls, and sharing invitations via WhatsApp groups facilitated by key neighbourhood figures. The researchers had no prior relationship with participants. Recruitment and interviewing took place between June and October 2023. The study focused on both birth parents and grandparents who acted as primary caregivers; hereafter, both are referred to as parents. Parents were invited to participate in the interview after providing their contact information. Families were counted as a single participant when both members of a couple participated. Recruitment faced challenges, including disinterest in being interrupted in public spaces, language barriers, reluctance to schedule follow-up interviews after a street conversation, and a lack of response to follow-up communication. However, all participants who agreed to participate completed the study.

### Data collection

The data collection involved semi-structured interviews. The interviews were conducted with parents and focused on exploring value tensions related to nutrition within families. Given prior research showing that *value tensions* can be an unfamiliar concept to participants [17], we used the term *difficult moments* in the interviews. This term encouraged participants to speak freely about difficulties they experience in their family regarding nutrition, while also covering value tensions.

During the interview, the daily *sensitisation exercises* were discussed as the main topics along with additional questions from our interview guide (Additional file 2: Table S2). Cards featuring words and images related to values, nutrition, and family life were used as prompts during the interview questions. These cards were used during the interview to help participants share their stories through associations with the words and images. This approach enabled deeper probing into their underlying knowledge and feelings about nutrition decisions, similar to the method by Sleeswijk Visser et al. [30]. The researchers developed these cards based on informal conversations with parents during recruitment and on existing studies on parents’ challenges, motivations, and considerations related to nutrition. For example, a theme found in literature called ‘Conflicts related to providing a snack just before dinner’ [17], led to selecting stock photos of a parent cooking and a set dinner table.

Questions about participants’ nutrition decisions for their children and experiences began with a discussion of the completed sensitisation exercises, followed by the use of the cards. If participants had not completed all exercises beforehand, the interviewer first explored their experiences openly, then used the cards to prompt further discussion. Laddering techniques were employed to uncover the underlying reasons behind difficult moments related to their children’s nutrition by asking questions such as, “Why was this moment difficult/important for you?” and “Can you provide an example of that?”. Next to the *why* question, which helped understand underlying values, participants were also asked to describe the difficult moment by focusing on the who, what, and when to understand the context. In addition to these open questions, the participants were also asked to provide demographic information during the interview, including their age, highest attained education, and work status.

The interviews, lasting between 60 and 90 min, were conducted in the participants’ homes or public locations like cafeterias, community centres or parks, depending on the participants’ preference. In most cases, children were present during the interviews. The interviews were conducted both individually and in pairs. All participants were offered the option to participate in a paired interview, which helped those who preferred being interviewed with a partner or friend feel more at ease. Data were collected through audio recordings and transcribed verbatim. Field notes were made immediately after each interview to capture initial insights and reflections. These field notes also showed that no new topics related to the research goals emerged in the final interviews, indicating that data saturation had been reached. Most interviews were in Dutch, except for two where either an interpreter was present or a translator app was used, along with image cards. Written informed consent was obtained from all participants. Participation was rewarded with a €20 gift card.

### Analysis

We conducted a thematic analysis [36], guided by the research objective of investigating difficult moments and related value tensions parents experience around nutrition. Our analysis combined inductive and deductive coding.

An initial familiarisation focused on parents’ challenges related to their child’s health and nutrition, yielding initial notes on their motivations, values, beliefs, and actions. Then, the first author and two researchers, familiar with the topic but not involved in the interviews, independently coded four transcripts through both open coding and deductive coding. For deductive coding, only the predefined concept of value tensions was applied. The researchers discussed and merged their codes into categories, with the first revealing *value tensions* related to child nutrition. The analysis of the open codes showed that both positive and negative factors contributed to these value tensions. These were grouped into two inductively defined categories: *stressors*, which triggered value tensions, and *protective factors*, which mitigated their impact. These categories served as the basis for a codebook used for subsequent interviews. Table 1 provides the three code categories and their definitions.

In later coding cycles, the first author coded additional transcripts using the codebook, refining categories collaboratively with the other researchers. The team iteratively reviewed and updated the codebook, adding codes and illustrative quotes. Code categories were then brought together into themes representing distinct value tensions, capturing key patterns in parental values that shaped nutrition decisions, and corresponding stressors and protective factors. Previously coded transcripts were also revisited to ensure alignment with the final coding scheme.

To enhance the interpretation of the themes, two final steps were taken. First, stressors and protective factors were categorised by their primary level of influence: child, parent, or community, drawing on the socio-ecological model [37, 38]. Second, classical content analysis was used to quantify the prevalence of value tensions by counting how many participants reported each type of value tension [39]. The unit of analysis was defined as each distinct instance of a nutrition-related value tension. The analysis was conducted using the Atlas.TI program [40].

To align with the research aims, we present only those tensions related to the value of *the dietary health of the child*. Additionally, some quotes revealed ambiguity, suggesting that multiple value tensions may coexist within a single situation. For example, parents seemed to use snacks when their child showed challenging behaviours to support their *well-being* or for *convenience*.

**Table 1:** Overview of final code categories and their definitions used to interpret parents’ experienced difficult moments around nutrition

Code categories	Definition
Perceived value tension	Values in tension are those that cannot both be fully satisfied during nutrition-related decisions. These value tensions underlie the *difficult moment*s parents experience when making nutritional choices for their child. Value tensions were inferred from actions, needs, and feelings, such as guilt or stress, that arose during *difficult moments*. To identify value tensions directly, we looked for phrases like “I would prefer to…, but…,” “I know it is not the best choice, although…,” or “I did…, however….” [17].
Stressor	Contextual constraint, a person or organisation causing the parent to experience a *difficult moment* and related value tension. For example, a child’s nagging for snacks before mealtime can act as a stressor to being able to satisfy both the dietary health of the child and the *enjoyment of the child*. The analysis focused on the parents’ perception of what caused the *difficult moment*.
Protective factor	Factors that help prevent or reduce the impact of stressors and tensions on parents. These factors exist both within and outside the family system. For instance, a babysitter can alleviate the stress of a crying child by providing support and reducing the parent’s exposure to the stressor. Importantly, *protective factors* exclude the parents’ actions, emphasising how external conditions can be structured to ease value tensions, shifting the focus from individual responsibility to the influential role of the broader environment.

### Researcher reflexivity

The research team consisted of three PhD candidates specialising in design, e-health, and medical ethics; two professors, one in human-centred design and the other in design for ecosystem innovation; and two research assistants with a background in social design. Interviews were conducted by three female members of the research team, with support from a fellow researcher who served as an Arabic interpreter during recruitment and one interview. None of the interviewers had a professional background in the medical field. Instead, their expertise lay in design research, with a focus on exploring human experiences rather than applying specialised knowledge in nutrition or stress. This orientation allowed the interviewers to engage with participants through empathetic listening and a curiosity-driven approach to understand lived realities. At the same time, the interviewers were outsiders to both the neighbourhood and the experience of caregiving, which may have led to missing nuances that those who had similar experiences would have intuitively understood. However, one interviewer had personal experience growing up in a disadvantaged neighbourhood, and two members of the broader research team are parents. These lived experiences added depth to the analysis. All of the team members involved in the analysis had relevant training in qualitative analysis. Throughout the study, the team remained mindful of how their backgrounds could influence assumptions and interpretations, engaging in continuous reflexive practice to ensure thoughtful and respectful engagement with participants’ lived realities.

## Results

### Participants

Eighteen families participated in the study, comprising 20 individual parents. Of these parents, two were grandparents living in the same household, acting as the primary parents, and therefore having responsibilities similar to those of birth parents. The study included 17 interviews, of which 14 were with individual participants. Three interviews were conducted with duos: two involving members of the same household and one involving parents from separate households. One participant did not meet the age criterion for children, as their child was over four years old. However, she was included because she could still reflect on her relevant past experiences with her five-year-old child, which aligned with the study’s objectives. Participant characteristics are summarised in Table 2.

**Table 2:** Participant characteristics

Participant characteristics	*N* = 20 (%)
Age	
Mean age	39 years
Median age	39 years
Age range	24–57 years
Not specified	2 participants (10%)
Gender	
Woman	18 (90%)
Man	2 (10%)
Child age (children 0–4 years old)	
Mean age	2 years
Median age	1.5 years
Age range	0–4 years
Child age (entire family)	
Mean age	5 years
Median age	4 years
Age range	0–15 years
Amount of children	
Mean amount	2 children
Median amount	2 children
Amount range	1–4 children
Highest level of education^1^	
Low education (ISCED 0–2)	5 (25%)
Medium education (ISCED 3–4)	2 (10%)
High education (ISCED 5–8)	10 (50%)
Not specified	3 (15%)
Job status	
Paid job	10 (50%)
No paid job	7 (35%)
Studying	1 (5%)
Not specified	1 (5%)
Marital status	
Single-parent household	4 (20%)
Two-parent household	16 (80%)

^1^International Standard Classification of Education 2011 [41]

## Value tensions

### Overview

Analysis of the interview data resulted in the identification of six value tensions underlying parents’ nutrition decisions, in which the health of their child was a central value. These are *(1) dietary health of the child vs. enjoyment of the child*,* (2) dietary health of the child vs. enjoyment of the parent*,* (3) dietary health of the child vs. convenience for parent*,* (4) dietary health of the child vs. affordability*,* (5) dietary health of the child vs. well-being of the parent*, and *(6) dietary health of the child vs. social belonging of the parent.*

The value tension between the dietary health of the child vs. enjoyment of the child played a role in at least one difficult moment for all participants, while the dietary health of the child vs. affordability contributed to difficult moments for the lowest number of parents (Table 3).

Each value tension will be addressed separately in the results section, together with the identified stressors and *protective factors* influencing these value tensions. An overview of all identified themes with quotes is available in the Additional files (Additional file 3: Table S3).

**Table 3:** The number of parents reporting each identified nutrition-related value tension

Value tension	Number of participants that mentioned tension (counting participants from the same family as one participant)
*Dietary health of the child vs. enjoyment of the child*	18 (100%)
*Dietary health of the child vs. enjoyment of the parent*	6 (33%)
*Dietary health of the child vs. convenience for the parent*	13 (72%)
*Dietary health of the child vs. affordability*	5 (27%)
*Dietary health of the child vs. well-being of the parent*	13 (72%)
*Dietary health of the child vs. social belonging of the parent*	9 (50%)

### Dietary health of the child versus enjoyment of the child

#### Perceived value tension

The value of a *child’s enjoyment* of food (Table 4) was influenced by parents’ perceptions of which foods their child liked and disliked. Often, the foods the child enjoyed were unhealthy, while healthier options were less appealing or were rejected. This created a value tension between parents’ value of *the child’s enjoyment* of food and the value of ensuring a healthy diet for the child.

**Table 4:** The value tension between the dietary health of the child vs. the enjoyment of the child, along with its stressors and protective factors

Tension origin	Stressors	Protective factors
Child	Child rejecting foods	The child enjoys healthy food
	Challenging behaviour of the child	Storage of healthy food alternatives
		An external person introduces food
		Food tips and validation from a peer group
Community	Treats at daycare & school	Daycare provides autonomy to the parent regarding treats
	Friends & family providing unhealthy foods	School policy is no treats

#### Underlying stressors

Parents experienced stress when children rejected food or displayed challenging behaviours around mealtimes. Signs such as shivering or spitting out food were perceived as dislike, and food rejection complicated making healthy meals that would satisfy *the child’s enjoyment* of food. Parents also struggled with their children having tantrums and nagging for unhealthy snacks, causing value tensions between limiting snacks for their *child’s dietary health* and valuing the *enjoyment* that snacks like candy can give their child.

*But secretly*,* you sometimes get really happy too. When they are happy*,* then you secretly also [feel happy]. You know it is [candy] not good*,* but still… (Participant 2*,* mother)*.

Participant three drew vital energy from their children’s *enjoyment*, gained through giving in to the stressor of their child’s nagging for snacks. Although it compromised *the dietary health of their child*, it helped them cope with the stress of solo caregiving, household responsibilities, financial strain, and frequent hospital and municipal appointments.

*Sometimes I feel just like a robot. I have to do so many things in a single day. How am I going to manage that? […] When are the children happy? […] You simply get energy from all of it. (Participant 3*,* mother)*

Community influences also incited value tension, as children were given unhealthy treats at daycare, school, or by family members. Removing the snack for the sake of *the child’s dietary health* would also mean taking away the associated value of *enjoyment*. For one parent, this meant accommodating her value of *dietary health for her child by her kids having crisps* once per week when her partner bought them, as it supported the *enjoyment* of her children.

*[I am] Never buying crisps. Only his father buys crisps. And it is enjoyable for the kids. But not so much [of the crisps]. Once per week. (Participant 4*,* mother)*

#### Protective factors

Several factors were identified that helped reduce the experienced stressors and following tension. Some children were perceived to enjoy healthy foods, reducing tension with the value of *enjoyment* of the child. Additionally, having healthy alternatives at home encouraged healthier snack choices during moments of nagging. While the parents’ social network could negatively impact them, it also helped lessen tension for two parents. Participant sixteen found support by sharing tips on healthy and enjoyable recipes for picky eaters with peers, which eased the tension by discussing food options that their picky eaters may enjoy. Additionally, sharing experiences with peers about her picky eater helped her feel validated as still being a good parent, reducing pressure consistently to fulfil both the *enjoyment* and *dietary health* of her child.

*For example*,* if you have a picky eater*,* you just want some ideas of what other picky eaters might easily eat. And then try that. Because I now have a few friends*,* and they also have picky eaters. And then*,* yeah*,* you just share a bit about who eats what. […] And sometimes you just want some support. Because at some point*,* you just do not know what to offer for dinner. […] So*,* a bit of that support*,* like*,* ‘Hey*,* you’re doing a good job as a mom.* (Participant 16, mother)

Furthermore, childcare workers and teachers contributed to exposing children to healthy foods. Participant eight shared a positive experience where collaboration with a childcare worker helped her child accept healthy foods, boosting her confidence in her child’s ability to enjoy them. This, in turn, reduced the value tension between her child’s *enjoyment* and their *dietary health*.

*I was really worried about it because he would gag every time he ate bread. He did the same thing at daycare. At some point*,* we [childcare worker and I] decided to keep offering it to him*,* so at least he would know what to expect. […] One day*,* I heard back from the childcare worker*,* ‘He ate the whole sandwich.’ Okay*,* great*,* fantastic. So that’s what I’m going to do now with fruit and everything else… I’m just going to keep offering it each time.* (Participant 8, mother)

Additionally, supportive policies reduced this value tension for some families by avoiding moments where children would nag for unhealthy treats. For instance, schools with “no unhealthy treats” policies and daycares that allowed parents’ autonomy over when to give treats helped mitigate pressure exerted by organisations in the community.

### Dietary health of the child versus enjoyment of the parent

#### Perceived value tension and underlying stressors

Parents experienced value tensions between the value of *enjoyment* enacted by their food preferences and the value of their desire to model healthy eating for their children. *Difficult moments* arose when children requested the same foods their parents ate, challenging them to balance personal *enjoyment* with setting a positive example (Table 5). For participant seven, this took shape as a tension between snacking during the day for their own *enjoyment* or considering the child’s *dietary health* by snacking at night.

*Yes*,* then he wants to copy me*,* of course. But you also have to set a good example yourself*,* right? I try to do it secretly*,* but sometimes it does not work. […] Even when they take an afternoon nap*,* I am snacking. And also when they go to sleep at night. (Participant 7*,* mother)*

**Table 5:** The value tension between the dietary health of the child vs. the enjoyment of the parent, along with its stressors and protective factors

Tension origin	Stressor	Protective factors
Child	The child wants food from the parent	-
Parent	Food preferences of the parent	-
Community	Friends & Family providing health tips	-

Parental preferences also influenced their child’s food decisions, with some parents avoiding healthy foods they personally disliked. This led to a value tension between promoting th*e dietary health of their child* and the *parents’ own enjoyment* of foods. Additionally, social influence, such as advice from family members, introduced tension between being conscious of nutritional concerns, like salt content, and the *parents’ enjoyment* of hearty, flavourful dishes.

*Because we really like savoury flavours. So we use a lot of spices. Until my mother-in-law once said*,* ‘You use a lot of salt.’ So then I started paying attention to it*,* also for the kids. (Participant 9*,* mother)*

No *protective factors* were identified in relation to this value tension. Since protective factors exclude the parents’ own actions, this may suggest that this value tension, centred on the *parents’ enjoyment* of food, is less susceptible to external influences and therefore harder to alleviate.

### Dietary health of the child versus convenience for the parent

#### Perceived value tension

The value of *convenience for parents* in tension with *the dietary health of the child* (Table 6) was reflected in quick, easy food choices. These food choices are not perceived as completely healthy, such as cookies, store-bought infant foods, or ordering take-out. Additionally, *convenience* was seen as using certain foods that are easier or quicker to cook than other options, but this often compromises *the child’s dietary health* due to their nutritional value.

**Table 6:** The value tension between the dietary health of the child vs. convenience for the parent, along with its stressors and protective factors

Tension origin	Stressor	Protective factors	
Child	Challenging behaviour of the child	**-**	
	Child rejecting foods		
Parent	Change of routine	Full availability for household tasks	
	Cooking time constraints	Informal- & formal household task support	
		Material & financial means	
Community	Undesired food choices in the neighbourhood	-	

#### Underlying stressors

Parents felt stressed when children’s nagging or crying disrupted tasks like cooking. This caused value tension between using sweets to quickly soothe their child for convenience and maintain healthy eating intentions. Food rejection by children was another stressor, especially when introducing solid foods or managing children’s refusal to accept certain foods. Participant eight felt insecure when her child rejected homemade meals, fearing they might not meet developmental expectations. She experienced tension in choosing store-bought infant foods for *convenience* and reassurance against choking, while she would have preferred homemade meals that she perceived as healthier. Regarding children rejecting foods, another parent found *convenience* in using sweets to encourage his child to eat more at dinner, but felt conflicted because he viewed treats as unhealthy and did not want to use them as a reward.

*Yes*,* I mean*,* if you are only eating cookies*,* sweets*,* and liquorice all day*,* that is obviously not really healthy food. It is more like a reward system. We do not use it as a reward system. […] If she has eaten enough*,* then she can have a candy in a bit. But I do try to push it off sometimes. […] A candy. Because I do not know… I said that it’s a bit easier. (Participants 9*,* couple*,* grandfather)*

Changes in routine, such as weekends or holidays, were mentioned by four parents and one couple as stressors that led to value tension between *convenience* and *dietary health for their child* in their food choices. Some parents made unhealthier choices on weekends, such as dedicating a day to eating fried foods. For others, it depended more on daily activities disrupting their healthy eating routine. They opted for *convenience* through quicker meals but compromised on nutritional value, often choosing less healthy options due to fatigue or limited time after caring for their children all day.

*If we have been in the park all day*,* then we go for something easier. You have been running after the kids the whole time. Then you think*,* I do not feel like cooking. (Participants 5*,* couple*,* father)*

Time constraints further exacerbated stress, particularly during busy work or study days or while managing all household tasks. Participant ten expressed feeling tension between their aspiration for home-cooked meals and the need to rely on convenient options such as a sandwich, due to time constraints from other caregiving responsibilities. While this more convenient meal was not inherently unhealthy, it compromised her ideal of a healthy meal.

*For example*,* I had two babies and I have little time. A baby from 0 to 6 months*,* I always find it difficult. Changing diapers every time*,* breastfeeding*,* sleeping*,* not going outside much*,* cannot always cook because [it] takes time. I just give my children a sandwich*,* spread it*,* it does not matter what. (Participant 10*,* mother)*

Additionally, environmental factors contributed to value tension. On busy days, parents found limited nearby food options that are both healthy and quick. This makes selecting nutritious meals more difficult and hampers their ability to prioritise *the dietary health of their child* fully. As a result, convenience is often prioritised to prevent late bedtimes, due to the restricted food options in the neighbourhood.

*So I really think that if I want to go get something*,* then here you can only choose from unhealthy options. […] But now I have the situation that I finish my internship at five o’clock. But yes*,* I have a one-year-old*,* so he needs to go to bed around seven o’clock. So at that moment*,* I would say*,* yes*,* you really should get take-out. (Participant 14*,* mother)*

#### Protective factors

Seven parents and two couples observed situations where the tension between their *child’s dietary health* and *convenience* was alleviated, highlighting *protective factors* that helped diminish this value tension. Firstly, in contrast to the stressor of time constraints from managing all household tasks, participant sixteen noted that having full availability for household tasks, rather than being a working mother, allowed them to prepare meals at more convenient times, thereby reducing reliance on unhealthy or inconvenient options.

*Then*,* before you pick up your child from school*,* you can quickly cook a meal. While you are hanging up the laundry*,* while you are keeping an eye on your little one. But look*,* if you are a working mother*,* either you have to batch cook everything*,* or you have to really buy things from the supermarket that you might not want at all. But for me*,* that has also been one of the reasons I said I am just staying home because I cannot keep up otherwise. (Participant 16*,* mother)*

Also, both formal and informal household task support, such as help from grandparents or daycare in providing meals or doing groceries, alleviated pressure on four parents regarding *convenience*, of which one was a couple. Daycare or school-provided healthy meals allowed parents to make convenient choices without feeling guilty during busy moments:

*And just like you said*,* eating healthy when you have a busy day. And if you eat fries once in the evening*,* they have eaten healthy all day at the daycare. (Participant 14*,* mother)*

Furthermore, access to resources such as freezers to store healthy, prepared meals for later use, or the financial means to buy or order healthy, convenient food options helped reduce or eliminate feelings of value tension.

### Dietary health of the child versus affordability

#### Perceived value tension

The value of affordability relates to parents’ financial stress when selecting food they consider healthy and affordable within their means during a tight budget. (Table 7).

**Table 7:** The value tension between the dietary health of the child vs. affordability for the parent, along with its stressors and protective factors

Tension origin	Stressor	Protective factors
Parent	Financial stress	-
Community	Daycare does not meet food wishes	Daycare provides mostly desired foods

#### Underlying stressors and protective factor

Four parents mentioned they have to cope with the financial pressure of rising food prices while trying to make healthy choices. Participant eight noted that the *affordability* of a product often outweighed its nutritional value when choosing between food brands.

*I prefer buying a cheaper brand of jam over a more expensive one. But I do not look at whether one has more sugars or fats than the other. (Participant 8*,* mother)*

This value tension was also felt by participant twelve, whose child attended daycare. The daycare provided meals, easing the parents’ worries about managing the *affordability* of healthy foods for their child.

*I just think*,* what is affordable? How can you do it? But of course*,* you want her to be healthy. And I am lucky that she also gets meals at daycare. So*,* in the contribution I pay*,* everything is included—food and diapers. (Participant 12*,* mother)*

However, the pescatarian diet provided by the daycare did not fully align with the parents’ view of a balanced, healthy diet. She decided to buy additional ingredients, such as meat, because she wanted her child to have them, despite the expense.

### Dietary health of the child versus well-being of the parent

#### Perceived value tension

The value of *well-being* for parents included the desire to experience rest, such as having time for themselves, and to avoid stress caused by children. It also involved feeling supported by others, especially their partner. However, *well-being* was found to be in tension with the value parents placed on their *child’s dietary health*. This value tension emerged when parents gave unhealthy foods to their child to soothe them and protect their *well-being*, or when they prioritised *the child’s dietary health* by preparing healthy meals at the cost of their own energy and time for rest (see Table 8). Although this conflict shares similar stressors with the tension between *the child’s dietary health* and parent *convenience*, it mainly focuses on promoting *the parent’s well-being* through nutrition decisions.

**Table 8:** The value tension between the dietary health of the child vs. the well-being of the parent, along with its stressors and protective factors

Tension origin	Stressor	Protective factors
Child	Challenging behaviour of the child	**-**
	Child rejecting foods	
Parent	No room for me-time	Routine
	Change of routine	
Community	Treats at daycare & school	**-**

#### Underlying stressors

A key source of tension between parental *well-being* and *children’s dietary health* was the child’s challenging behaviour, such as nagging, crying, or disobedience, reported by nine parents. To cope, parents often used snacks to manage these behaviours and reduce their own stress, especially while cooking or when feeling unwell. When children persistently asked for unhealthy foods like candy, giving in would stop the nagging, even if it conflicted with the parents’ values around healthy eating. In addition to food, two parents also used screens to ensure uninterrupted time for cooking or eating. For instance, participant ten, despite emphasising the importance of limiting sugar intake, used sweets as a reward for obedience to avoid conflict when feeling tired or sick.

*First*,* I focus on the food. I give them what is healthy*,* less sugar. […] If I am tired or sick… They do not listen as strictly anymore. I take a break and calmly talk to them. I say*,* ‘If you listen and behave well for mom*,* then [you can have or do] whatever you want…’ They usually want to go outside or have a treat*,* like chocolate or something. (Participant 10*,* mother)*

Another stressor was children rejecting foods, which overwhelmed parents already dealing with work-related stress. Participant thirteen described feeling so mentally drained that she could not deal with the rejection of the food she had cooked for her child. Consequently, it became challenging for her to ensure her child would eat after a busy day while also caring for her own *well-being*.

*When work is busy or you have still got things on your mind*,* and then you come home and there is chaos everywhere with toys and everything. And then you think*,* oh right*,* I still need to deal with food*,* and you make something*,* and suddenly they want something else*,* and yeah. I just do not have the mental space for it then. (Participant 13*,* mother)*

Parents’ stress was also connected to their lack of personal time. Two parents described a tension between preparing meals for their children for their *dietary health*, and participating in activities like community breakfasts at the neighbourhood centre to support their *well-being*. As a result, their ability to focus on their *well-being* was reduced due to childcare demands, such as providing healthy food.

*Participant 3: I used to [eat breakfast with women from the neighbourhood]*,* but now with the kids. Now I have to go back to give them a sandwich. I am busy. So*,* that is why.*

*Participant 2: Before*,* I was not so busy. When I did not have a baby*,* I eat nice breakfast here. It was cosy.” (Participant 2 & Participant 3*,* mothers)*.

Additionally, parents used snacks to boost their *well-being* on stressful days, causing a feeling of tension with setting a healthy example for their children:

*If I have had a hard day at work*,* I think*,* well*,* I can have that extra cup. Then I think*,* yeah*,* actually it is a bit of a double standard. What you teach your child*,* you are not doing yourself. Really*,* I should be setting an example. I want them to have a regular routine. I want them to eat healthy. Then I should be doing that for myself too. (Participants 5*,* couple*,* mother)*

Additionally, a change in routine caused tension. Bedtime disruptions due to healthy dinners being prepared too late took a toll on *parents’ well-being*, as it increased challenging behaviour in their children the next day. Finally, treats at daycare also caused tensions regarding *parents’ well-being*. Participant fifteen mentioned that her children become too energetic in the evening because of treats brought home from daycare or school. This stressor is also related to the value tension of the *enjoyment of the child*, while this tension mainly focuses on protecting the *parent’s well-being* rather than taking into account the *child’s enjoyment* of treats.

#### Protective factors

While daycare and school had adverse effects due to treats, the routine of the eldest children going to school acted as a *protective factor*. It allowed parents time for both self-care, such as meeting up with a friend, and household tasks like cooking.

*Routine is really important in life. […] The kids go to school. You get back to what you are doing. Eat. Make food. Clean up. Pick up the kids. Eat. Have some playtime. Bedtime. […] Routine with your friend. You can have coffee together. (Participant 3*,* mother)*

### Dietary health of the child versus social belonging of the parent

#### Perceived value tension

The final value tension in *dietary health of the child* vs. s*ocial belonging of the parent* concerns tensions parents experience in adhering to norms regarding food for their child within their social circle. This created a sense of difficulty maintaining the desired connection with partners, grandparents, and friends, while they would endorse foods perceived as unhealthy for their child (Table 9).

**Table 9:** The value tension between the dietary health of the child vs. the social belonging of the parent, along with its stressors and protective factors

Tension origin	Stressor	Protective factors
Child	Child rejecting foods	-
Community	Friends & Family providing unhealthy foods	The child eats little of the snack
	Lack of shared health values with other parents	Others accept the parents’ food rules
		Role model in social circle

#### Underlying stressors

Children’s food rejection caused tension around feeding. Participant eight expressed insecurity when their child fell behind peers in accepting solid foods, showing a need to conform to social standards of child health. At the same time, this was in tension with what they later perceived as actually important for *their child’s dietary health*:

*I secretly think sometimes*,* like*,* ‘Oh*,* that child is the same age as mine. They are already eating the same meals as the family*,* even things like paella with all sorts of ingredients. But mine is not doing that yet.’ I have to admit*,* I found that a bit difficult at first. […] I think it had more to do with my own insecurities than with it being important for him to eat the same meals as the family (Participant 8*,* mother)*.

At the community level, seven parents experienced stressors related to social influence from friends and family. Grandparents and other family or friends often offered children foods considered unhealthy during visits, parties or while babysitting. Participant sixteen shows how visits to the children’s grandmother create tensions between her usual healthy rules for her children and the social significance of the visit. During these times, unhealthy snacks are allowed to support *social belonging* and stay connected with the grandmother.

*So yeah*,* she has M&Ms on the table. […] But I just let it go because they do not see their grandmas that often. […] So it is really negligible. If they want to snack on sweets*,* cakes*,* and whatever else. (Participant 16*,* mother)*

Next to grandparents, partners also had influence. They could hold different opinions about what constitutes good *dietary health* for their child. Three participants mentioned disagreements with their partner over how to handle their child’s refusal to eat certain foods or about having snacks in the house. Finally, participant twelve reported feeling isolated because of a lack of shared *dietary health* values with other parents, which hinders meaningful connections. This created a value tension between her child’s *dietary health* and conforming to social norms of parents around them for *social belonging*.

*It is not like you really build friendships there [at community centre meet-ups]*,* or that you get more personal contact. […] And I think maybe also because of the things I’ve been through in life*,* and because I am older*,* I do not approach life as casually anymore. […] They [other parents] easily give their kids a cookie or put them in front of YouTube. Yeah*,* they just handle things very differently.* (Participant 12, mother)

#### Protective factors

Participant seven found it less challenging when others offered snacks because her child ate little of the snack. Respect for the parents’ food rules from their social environment also eased the value tension. Additionally, having a role model for healthy eating in her social circle helped another participant address both *the dietary health of her child* and her feeling accepted by someone in her social circle, satisfying the value of *social belonging*. Her sister-in-law introduced healthy alternatives earlier in life, rather than the unhealthy norms of her mother and culture.

*She [sister-in-law] always said*,* ‘Yeah*,* you should not give sugar until they are two years old; it is not good.’ My mom*,* on the other hand*,* was more into giving sugar. For example*,* honey*,* which is actually forbidden for children under one year old. But in our culture*,* we just give it to every child*,* letting them taste it or something. Or they put chocolate on a pacifier to get the child used to it*,* you know? She [my sister-in-law] was always against it. And when she had her second child*,* I was already 15 or 16 years old*,* so I saw it all happening from her. (Participant 7*,* mother)*

## Discussion

### Principal findings

The current study aimed to provide insights into the value tensions that parents in disadvantaged neighbourhoods in the Netherlands face in making nutrition decisions for their children. We identified six value tensions related to the nutrition decisions parents make for their children, along with the stressors that cause them. Additionally, we identified *protective factors* that diminish the impact of experienced stressors, such as informal and formal household task support, routine, and non-family members introducing foods. Our generated insights contribute to the current knowledge of the various factors that influence parents’ food decisions for their children. While these value tensions are probably felt by parents more generally, our findings indicate that managing them may be more challenging in disadvantaged settings, where overall stress levels are higher. Resources to reduce stressors can be more limited due to socioeconomic barriers. The findings help guide the creation of nutritional interventions targeted at parents in disadvantaged settings, who face the most significant difficulties in making healthy nutrition choices for their children.

### Main value tensions

The three most prevalent tensions experienced by participants were Dietary health of the child vs. enjoyment of the child, Dietary health of the child vs. convenience for parent, and Dietary health of the child vs. well-being of the parent. These tensions reflect the complexity parents face in making healthy food choices for their child when (1) facing the child’s dislike of certain healthy foods or not wanting to take away enjoyment of unhealthy foods, (2) finding convenient options during busy and stressful moments, and (3) providing unhealthy foods to reduce stress or preparing healthy meals at the expense of the parents’ well-being. Previous studies have also indicated that concerns about enjoyment, the influence of others, time, and convenience play a role in parents’ food choices. These findings apply to parents of children aged 2–7 and those in disadvantaged neighbourhoods with children under 18 years old [17, 27]. Other studies have also shown that parents cope with work stress and psychological distress by indulging in treats or opting for convenient foods [19, 27, 42]. Our findings elaborate on this by identifying specific stressors and moments that impact healthy nutrition decisions concerning the value tensions parents experience. For example, we show that when children exhibit challenging behaviour by persistently requesting snacks at home, it can create stress for parents. This stress stems from the emotional discomfort of seeing their child unhappy, leading them to give in to the request to meet the value of their child’s enjoyment, at the expense of the child’s dietary health.

### Tailor interventions to specific value tensions to support healthy nutrition

Our study highlights a key value tension between *parent well-being* and *child dietary health*, which underlies at least one challenging moment for most participants. Therefore, we propose that stress management is crucial for parents to make healthier food choices for their children, consistent with research by Jang et al. [19]. This also incorporates recognising parents’ *reasonable responses* to preserve their *well-being* and other values in the moment, such as offering snacks to calm their children. These nutrition decisions are *reasonable* within the constraints and benefits of their context [28]. This includes time pressure and limited household support, where snacks help parents manage stress and complete household tasks, such as preparing a healthy meal. This dynamic may be especially pronounced in parents living in disadvantaged neighbourhoods, who have a higher risk of perceived stress and associated unhealthy nutrition decisions [43, 44]. Additionally, our results indicate that parents can draw significant energy from their *children’s enjoyment*, which snacks can spark. This was a strength for one participant, dealing with depleted energy and stress due to being a single parent, financial constraints, and time pressure caused by frequent municipal appointments for social services. Through understanding these contextualised nutrition decisions, interventions can be attuned to the factors that undermine healthy eating for families [45]. Interventions, local parent activities, and routine care should address underlying tensions for specific challenging moments around nutrition. For example, providing care tips that balance *the dietary health of the child* and the parents’ *well-being* during dinner, when children may demand attention or nag for snacks. This also holds for the other identified value tensions.

Additionally, our findings reveal overlapping stressors across value tensions, demonstrating that the same challenge, such as a child’s difficult behaviour, can be experienced differently depending on the parent’s values and context. This highlights that there is no one-size-fits-all solution, as parents require tailored support based on their unique situations. For instance, giving a child a snack in response to tantrums may reflect a desire to preserve the c*hild’s enjoyment*, a convenient way to soothe them, or a moment for the parent to regain calm to promote their own *well-being*. Intervention designers should consider offering diverse strategies to help address different underlying values in tension. These can include suggestions for enjoyable, healthy snack alternatives or local free and fun child-friendly activities as a substitute source of enjoyment to foods (*enjoyment*); Improved access to convenient, nutritious snacks or a program with household support to ease multitasking (*convenience*); and support for family routines through access to affordable childcare, which creates time for the parent to recharge (*well-being*). These strategies need to be combined, as one value can be prominent while others still influence the parents’ nutrition decision.

### Strengthening community-based protective factors to support healthy nutrition

While most parents experienced value tensions as continuously *difficult moments*, we found that some parents experienced *protective factors* that lessened the tension or made it disappear entirely. We speculate that parents across all socioeconomic strata experience the same identified value tensions, as the underlying stressors are not unique to any one group. Indeed, several studies have shown that parents can face multiple stressors when making healthy nutrition decisions for their young children, including time constraints, picky eating, frequent requests for snacks, child tantrums, unhealthy parental food preferences, and the influence of other adults such as partners, extended family, and childcare providers [17, 46–48]. We contribute to this literature by showing that the extent to which these stressors and underlying value tensions are felt likely depends on the availability of *protective factor* resources, which reduce the perceived intensity of these stressors on nutrition decisions. Thus, protective factors would make it easier to cope with value tensions. This is supported by Bukman et al. [49], who found that participants with a low socioeconomic position viewed their current lifestyle as a logical adaptation to their situation and constraints, highlighting the need to address contextual factors alongside individual choices. Given the nature of the *protective factors* discussed, such as social and material resources, we expect that people living in socioeconomically disadvantaged contexts, like the disadvantaged neighbourhoods in our study, have fewer protective factors available to cope with value tensions than other groups.

Social support, including household task support and material support, such as the means to obtain alternative, quick, and healthy meals, was a critical *protective factor* for participants in our study. Social support in cooking and food preparation has also been identified by Jang et al. as supporting healthy nutrition practices and reducing stress in families [19]. Thus, interventions targeting only individual behaviour change fall short if they do not address the social determinants of health inequity [50, 51], such as poverty, housing, and isolation, which, in turn, affect the availability of resources for protective factors. Current interventions often fail to align with these lived realities and the deeper motivational factors shaped by social, cultural, and economic contexts to achieve behaviour change [52]. We propose that tailoring interventions to address individual value tensions, alongside community approaches such as strengthening social support, helps parents navigate these value tensions and ultimately reduces health disparities. Increasing social support is especially promising, as socioeconomically disadvantaged people are more likely to experience low social support [53], while social support is a promising means of promoting health and healthy nutrition [4, 54–56]. Although some participants in our study demonstrated strengths in both receiving and providing social support around nutrition within their communities, this was true for only a small number of them.

P*rotective factors* were more prevalent in navigating the tension between *the child’s dietary health* and *convenience* than in the tension between *the child’s dietary health* and *parental well-being*, which only had the *protective factor* of routine. This result indicates that parents can already make more accessible and *convenient* choices, rather than choices that support both the *child’s dietary health* and the *well-being of the parent*. Therefore, interventions need to support the identification of possible *protective factors* as well as enable the availability of *protective factors* to lessen this value tension. For example, an intervention can involve a community-based platform that connects parents to prepare meals using shared ingredients collaboratively. This approach offers material and instrumental support by reducing individual costs and effort, while also strengthening informal care networks. Neighbours or parents who become trusted through cooking together can, for instance, step in to help with childcare during busy moments, creating a flexible and supportive care system. Therefore, we suggest that intervention designers include protective factors, such as social support, in their interventions by enhancing existing factors or helping families develop them. Policymakers could address the social determinants of health, such as economic stability, access to healthy foods, and social cohesion, to improve parents’ access to *protective factors*. We propose that public health efforts integrate both approaches: reducing daily stressors that influence parents’ nutrition decisions and tackling structural barriers. This includes supporting protective factors such as family routines, household tasks, and access to healthy meals through childcare, while also addressing broader systemic issues that limit access to these supports [29]. Together, these efforts can create the conditions that enable parents to make healthier nutrition decisions for their families.

### Coping with values simultaneously

Lastly, while protective factors reduced some value tensions, many parents reported unresolved value tensions. This finding is supported by Devine et al. [42], who found that working parents with low income mainly set priorities among nutrition-related goals to manage stress, rather than addressing the source of the tension. For example, parents faced stress from underlying issues, such as limited time due to work demands and needing to earn sufficient income. They often prioritised quick meals to manage immediate needs over healthier options that support long-term dietary health. While this is a reasonable response, neglecting dietary health led to feelings of guilt and stress among parents, as shown in both Devine et al. [42] and our findings. Therefore, future studies should investigate how parents can cope with value tensions so that both values supporting the present (e.g., enjoyment of the child) and the future (e.g., dietary health of the *child)* are addressed, reducing negative affect. A promising lens for such studies is Paradox Theory, which focuses on managing values in tension simultaneously [14]. It is extensively studied and applied in management literature, and is also gaining relevance in health contexts. For instance, Yan [57] discusses their perspective on the value tension between health and enjoying leisure when it comes to bedtime procrastination, proposing a paradoxical approach that includes solutions like balancing these values by redistributing leisure time during the day.

### Limitations

The results offer new insights into the value tensions and influencing factors shaping parents’ experiences with nutrition decisions. However, several limitations should be discussed. First, response bias may have influenced the findings, as nutrition decisions were self-reported. Parents often desired to be seen as ‘good parents’, potentially leading to socially desirable responses. For example, one parent stated they avoid giving their child sugar for health reasons, but later mentioned offering sugary snacks to stop their child from whining while cooking. Such discrepancies may also stem from parents’ unawareness that their actions and underlying values are in tension. Another potential limitation is using cards with words and images as prompts during interviews. While meant to support reflection and engagement, they may have narrowed the discussion by steering participants toward predefined themes, possibly limiting novel insights. To mitigate this, interviews started with open-ended questions and sensitisation exercises, and cards included both specific and broad images and words. In one interview, a translator app was used for communication, which could have affected the depth of the responses and transcription accuracy. Despite this, visual prompts and pre-prepared cards aimed to support understanding. Differences in interview formats, whether conducted with pairs or individuals, may have influenced how openly participants discussed sensitive topics such as stress or disagreement. Nonetheless, these topics were covered in both individual sessions and interviews with two participants. Additionally, the identified value tensions were not validated by participants and should be interpreted as researchers’ analyses. The study focused on nutrition and related stress, excluding behaviours such as physical activity and screen use unrelated to nutrition, which some parents also discussed as moments they found difficult. This indicates that value tensions can be more pronounced in other health areas depending on parents’ perceptions of health and currently experienced stressors. Future research should prioritise the needs of parents to adapt the research focus to their most pressing concerns. Finally, recruitment challenges may have introduced bias and limited generalizability. Participants may have been more inclined toward health-related topics due to the study’s focus. To mitigate this, we recruited parents in public spaces such as streets, aiming to engage those not already involved in parenting or child health activities at venues like community centres. However, since most recruitment occurred during daytime hours, this may explain the limited participation of fathers in our study due to full-time work obligations. We recommend that future studies actively involve fathers in ways that suit them, as this may help uncover additional value tensions within families. Additionally, our sample includes a relatively high proportion of participants who completed tertiary education, classified as ‘high’ education, which may have influenced our findings. This may be slightly skewed, as three participants did not report their education level. Still, participants were recruited based on their neighbourhood to avoid stigma and emphasise how social and structural factors affect health behaviours, rather than just personal characteristics like education.

## Conclusion

This interview study highlights the complexity of value tensions underlying parents’ nutrition decisions in disadvantaged neighbourhoods. Key tensions, balancing the value of the *child’s health* with *convenience*, *parent well-being*, and *social belonging*, reveal how competing demands shape parents’ food decisions for their children. Although scarce, some parents experienced reduced value tension regarding *the dietary health of their child* through *protective factors* such as social support (e.g., informal childcare) and material support (e.g., ingredients for quick, healthy meals). The limited presence of resolved value tensions through protective factors highlights the challenge of insufficient resources to address these tensions, further supporting evidence of how socioeconomic health inequities affect nutrition decisions. These findings suggest that while parents want to offer healthy food for their children, the stressors identified in this study and structural barriers can make it challenging to do so in practice. We recommend tailoring nutrition interventions for parents of children aged zero to four to address the value tensions they face and the moments when these tensions occur. This means that interventions cannot focus solely on the value of *dietary health*, like in information-based interventions, as this would keep the value tension and related stress intact. Additionally, interventions are recommended to address the social and structural environments of parents by incorporating social and material support within the community and tackling interconnected health inequalities, as they may alleviate stressors that cause value tensions in the first place.

## Supplementary Information

Below is the link to the electronic supplementary material.

Supplementary Material 1

Supplementary Material 2

Supplementary Material 3

## Data Availability

The datasets used and/or analysed during the current study are available from the corresponding author on reasonable request.
