# Supplementary material for: How tensions between parents’ values influence decisions about their children’s nutrition: a qualitative study in disadvantaged neighbourhoods
Source: Int J Equity Health. 2025 Dec 5;25:6. doi: 10.1186/s12939-025-02712-y (PMC12797942; doi:10.1186/s12939-025-02712-y)
Supplement: Supplementary file 1 — Supplementary Material 1 [file 12939_2025_2712_MOESM1_ESM.docx]

**Additional file 1: Table S1.** Sensitizing exercise topics and exercise description

| **Topic** | **Exercise description** |
| --- | --- |
| Family structure | Describe what your family looks like In this first task, I would like to learn about who lives in your household. Would you be able to send me a photo of your family? You can also describe your family using emojis. After this, I will send you a few more questions about your family.  Who is in the photo? / Who lives in your household? What are their ages?  What do you do in daily life?  Besides the people in your household, are there others involved in raising your child? Who are they, and what role do they play in your child's upbringing? |
| Nutrition habits | Describe what your family eats, drinks, when and with whom  In this task, I would like to learn more about what your family eats—specifically what you, your partner (if applicable), and your child or children aged 0-4 eat.  Would you be able to take and send photos of what your family eats in a day? You can include moments such as breakfast, lunch, snacks, and dinner. |
| What is important to you around nutrition | Describe what your family pays attention to regarding nutrition  In this task, I would like to learn more about what you pay attention to regarding nutrition in your family. You can send a short message or voice recording about what you focus on when it comes to your family's nutrition.  You may include:   - What you pay attention to regarding food and drinks for your child. - What you do not pay attention to regarding food and drinks for your child.   As inspiration, you will receive an image with example words, and in this video, I will mention a few. For example, you might think about price, enjoyment, convenience, energy, and safety. |
| Difficult moments | Describe difficult moments related to nutrition in your family  In this task, I would like to learn more about difficult moments during the day related to nutrition in your family. These moments could be when you feel pressured, irritated, stressed, or uncertain.  Could you send these moments in a message or voice recording? You may describe moments that have happened in the past year or ones you experience today. |
