# Supplementary material for: How tensions between parents’ values influence decisions about their children’s nutrition: a qualitative study in disadvantaged neighbourhoods
Source: Int J Equity Health. 2025 Dec 5;25:6. doi: 10.1186/s12939-025-02712-y (PMC12797942; doi:10.1186/s12939-025-02712-y)
Supplement: Supplementary file 2 — Supplementary Material 2 [file 12939_2025_2712_MOESM2_ESM.docx]

**Additional file 2: Table S2.** Interview topics and example questions

| **Topic** | **Example question** |
| --- | --- |
| **General/ Family structure** |  |
| Sensitizing exercises | What are your thoughts on the tasks you received through WhatsApp? |
|  | How did filling out the tasks go for you? |
| Household composition | Who do you live with? |
|  | What are the ages of you and your family members? |
| Demographics | What do you do in your daily life? (Occupation/school/home) |
|  | What level of education have you and your family members completed? |
| Support | Do you have people around you who supports you? |
|  | How do they support you? |
|  | Do you experience support in taking care of your child? |
| **Nutrition habits** |  |
| Own nutrition | What did you eat on the day of the task [refer to preparatory task to take photos of what was eaten]? |
|  | Could you explain to me what is shown in the photos? |
|  | Is this something you eat regularly, or was it an exception? If it was an exception, what do you normally eat? |
| Child’s nutrition | What did your child/children eat on the day of the task [preparatory task to take photos of what was eaten]? |
|  | Could you explain to me what is shown in the photos? |
|  | Is this something you eat regularly, or was it an exception? If it was an exception, what do you normally eat? |
| Meal preparation | How often do you cook per week? |
|  | How often do you get takeout or eat out per week? What do you get? |
|  | How often do you have ready-made meals per week? What do you eat? |
| Special occasions | What do you eat on special occasions like parties or on weekends? |
|  | How do they (reference to previous question) affect what you eat? |
| **What is important around nutrition** |  |
| Important aspects of nutrition | What do you pay attention to regarding food and drinks for your child? [*Refer to words chosen regarding what the participant pays attention to around nutrition in preparatory exercise + additional cards with words to probe during interview*] |
|  | Why do you consider that important? |
|  | Can you provide an example? |
| Aspects of lesser attention or unimportance around nutrition | What do you not pay attention to regarding food and drink for your child? [*Refer to words chosen regarding what the participant does not pay attention to around nutrition in preparatory exercise + additional cards with words to probe during interview*] |
|  | Why do you consider that less important? |
|  | Can you provide an example? |
| Difference between points of attention for child and parent | Are there things you pay attention to for your child but not for yourself? |
|  | Are there things you don't pay attention to for your child but do for yourself? |
| **Difficult moments** |  |
| Nutrition in family/for the child | What are difficult moments regarding food in your family [refer to moments written down in preparatory task] ? |
|  | Why is this moment difficult for you? |
|  | Who else plays a role in this moment? |
|  | How do you handle such moments? |
|  | How do you feel about such moments? |
|  | Can you provide examples? |
|  | How often do these kinds of moments occur? |
| Own nutrition | What are challenging moments regarding your own nutrition? |
|  | Why is this moment difficult for you? |
|  | Who else plays a role in this moment? |
|  | How do you handle such moments? |
|  | How do you feel about such moments? |
|  | Can you provide examples? |
|  | How often do these kinds of moments occur? |
| Difficult moments when stressed | What are difficult moments when you feel stressed or overwhelmed? |
|  | Why is this moment difficult for you? |
|  | Who else plays a role in this moment? |
|  | How do you handle such moments? |
|  | How do you feel about such moments? |
|  | Can you provide examples? |
|  | How often do these kinds of moments occur? |
| Handling difficult moments | What are you proud of in how you handled difficult situations related to nutrition? |
| **Closing topics** |  |
| Future | What would you want do differently in the future? |
|  | What would you need for that? |
| Recommendations | What are you proud of in your family that you would recommend to other families as well? |
|  | Do you have any further tips for other families? |
| Missed topics | Is there anything you would like to add that I didn’t ask about? |
