# Supplementary material for: How tensions between parents’ values influence decisions about their children’s nutrition: a qualitative study in disadvantaged neighbourhoods
Source: Int J Equity Health. 2025 Dec 5;25:6. doi: 10.1186/s12939-025-02712-y (PMC12797942; doi:10.1186/s12939-025-02712-y)
Supplement: Supplementary file 3 — Supplementary Material 3 [file 12939_2025_2712_MOESM3_ESM.docx]

**Additional file 3: Table S3.**Table of themes, sub-themes, and codes with supporting quotations

| **Themes** | **Sub-themes and codes** | **Quote** |
| --- | --- | --- |
| Dietary health of the child vs the enjoyment of the child | Stressors - Child rejecting foods | "Well, yeah, when they say they like something and then you make it, and suddenly… they say they do not like it anymore, just like that. Especially the youngest, really, but that… yeah, I find that difficult. I mean, last week you thought it was amazing, and now..." (P13, mother) |
|  | Stressors - Challenging behaviour of the child | "Look, it happened this afternoon too. It was when they came home and said, ‘Grandma, can I have a candy?’ Then I think, well, you have not had much candy today. I get the feeling she hardly gets any sweets at daycare. So I give her two candies. And then she says, ‘They were really tasty. Just one more.’ Then I say, no, now you are not getting any more. You have had enough for today. That is it. And sometimes she agrees with that. And other times she throws a tantrum." (P20, couple, grandmother) |
|  | Stressors - Treats at daycare & school | I noticed — my oldest also went to this preschool — that they are a bit less strict with the youngest. But we are still strict about it. Sometimes in the summer they hand out ice creams. We always send our own ice cream, just homemade from juice. (P13, mother) |
|  | Stressors - Friends & family providing unhealthy foods | "I do find that difficult sometimes. Because she sometimes comes with ice creams before dinner, for example. But because we talk about it openly, she always comes to me with the ice cream. And then she says, 'Mama, I got this. May I eat it?' No. Because we are about to eat. Put it in the freezer and then maybe you can have it as dessert. Or the next day. Or when she comes with a bag of candy just before dinner. Then I say, yes, you know, not now. And also in the afternoon with a bag of candy that she got from someone." (P8, mother) |
|  | Protective factor - Child enjoys healthy food | "Because when a child is used to candy from a young age, then their child will want that. But mine are not used to it, they do not like it. And then, yes, she finds it tastes very strange, so to speak."(P7. mother) |
|  | Protective factor - Storage of healthy food alternatives | "Then I do not know anymore what you are supposed to give. I keep trying to offer variety. I do not want to give fruit all day long, for example. But at a certain point, you run out of options. Unless you have a pantry with 5,000 different things. So at some point, you just do not know what to give anymore. Eventually, the bell peppers come out. And then it is bell peppers to eat. That is healthy then." (P5, couple , father) |
|  | Protective factor - An external person introduces food | "Because a few months ago, he would not even eat bread. I was really worried about it because he would gag every time he ate bread. He did the same thing at daycare. At some point, we [childcare worker and I] decided to keep offering it to him, so at least he would know what to expect. If he did not want it, that was fine, but at least we tried. And you notice that the more you do that, eventually it changes. One day, I heard back from the childcare worker, 'He ate the whole sandwich.' Okay, great, fantastic. So that’s what I’m going to do now with fruit and everything else… I’m just going to keep offering it each time." (Participant 8, mother) |
|  | Protective factor - Food tips and validation from a peer group | "For example, if you have a picky eater, you just want some ideas of what other picky eaters might easily eat. And then try that. Because I now have a few friends, and they also have picky eaters. And then, yeah, you just share a bit about who eats what. [...] And sometimes you just want some support. Because at some point, you just do not know what to offer for dinner. And what I would recommend is just to offer it. And if they do not want it, give them a peanut butter sandwich or something. But it only goes so far. So, a bit of that support, like, ‘Hey, you’re doing a good job as a mom.” (Participant 16, mother) |
|  | Protective factor - Daycare provides autonomy to the caregiver regarding treats | "Yes, you get those in a little bag. Or sometimes they just put it in the lice bag. They often get those toddler cookies, baby cookies. Sometimes there are candies in there, but sometimes there are also little gifts, like pencils and a coloring page, different little things. She does not get the candies and cookies. I keep those. And we will see when or how or what. Definitely not if it is really candy candy. But if it is those toddler cookies, for example, then I do keep them." (P12, mother) |
|  | Protective factor - School policy is no treats | "Yes, it means that the children are not allowed to bring juice to school. That rule was introduced about two years ago or so. With a new principal. So they are not allowed... So they are not allowed to bring juice boxes. They are not allowed to bring treats. So no cookies and such. They have to bring healthy treats." (P15, mother) |
| Dietary health of the child vs enjoyment of the caregiver | Stressor - Child wants food of the caregiver | "Yes, then he wants to copy me, of course. But you also have to set a good example yourself, right? I try to do it secretly, but sometimes it does not work. [...] Even when they take an afternoon nap, I am snacking. And also when they go to sleep at night." (Participant 7, mother) |
|  | Stressor – Food preferences of caregiver | Yes, actually I thought, yes, you do like it. So do not complain. But I cannot really argue it. Because I do not eat it either. Then you also cannot really make the argument that it is tasty. Or that you should just eat it. (Participant, 6) |
|  | Stressor - Friends & Family providing health tips | "Because we really like savoury flavours. So we use a lot of spices. Until my mother-in-law once said, 'You use a lot of salt.' So then I started paying attention to it, also for the kids." (Participant 9, mother) |
| Dietary health of the child vs convenience for the caregiver | Stressor - Challenging behaviour of the child | “And usually when I'm cooking, they are both standing there. [...] They cannot wait for the food. And that's the only thing I struggle with—that they cannot wait. But yeah, it's done quickly. But in the meantime, I sometimes give them a cookie, and then they keep waiting there. [...] When they are eating, I also give them a cookie. Then they are quiet, too. Then I keep giving those cookies.” (Participant 7, mother) |
|  | Stressor - Child rejecting foods | "Yes, I mean, if you are only eating cookies, sweets, and liquorice all day, that is obviously not really healthy food. It is more like a reward system. We do not use it as a reward system. [...] If she has eaten enough, then she can have a candy in a bit. But I do try to push it off sometimes. [...] A candy. Because I do not know... I said that it’s a bit easier." (Participants 9, couple, grandfather) |
|  | Stressor - Change of routine | "If we have been in the park all day, then we go for something easier. You have been running after the kids the whole time. Then you think, I do not feel like cooking.” (Participants 5, couple, father) |
|  | Stressor - Cooking time constraints | “For example, I had two babies and I have little time. A baby from 0 to 6 months, I always find it difficult. Changing diapers every time, breastfeeding, sleeping, not going outside much, cannot always cook because [it] takes time. I just give my children a sandwich, spread it, it does not matter what.” (Participant 10, mother) |
|  | Stressor - Undesired food choices in the neighbourhood | “So I really think that if I want to go get something, then here you can only choose from unhealthy options. [...] But now I have the situation that I finish my internship at five o'clock. But yes, I have a one-year-old, so he needs to go to bed around seven o'clock. So at that moment, I would say, yes, you really should get take-out.” (Participant 14, mother) |
|  | Protective factor - Full availability for household tasks | "Then, before you pick up your child from school, you can quickly cook a meal. While you are hanging up the laundry, while you are keeping an eye on your little one. But look, if you are a working mother, either you have to batch cook everything, or you have to really buy things from the supermarket that you might not want at all. But for me, that has also been one of the reasons I said I am just staying home because I cannot keep up otherwise." (Participant 16, mother) |
|  | Protective factor - Informal- & formal household task support | “And just like you said, eating healthy when you have a busy day. And if you eat fries once in the evening, they have eaten healthy all day at the daycare." (Participant 14, mother) |
|  | Protective factor - Material & financial means | "Yes, actually I do everything by public transport. And with two children, that is quite inconvenient. So I usually just get whatever I think, oh, they will have this. And then I buy that. For example, here at the supermarket, they have a lot of vegetables and such. And fresh. So I often get vegetables and eggs there, those kinds of things. And usually when my partner is around, then we go by car and then I get everything for the whole week. Then I put it in the freezer and see how it goes." (P14, mother) |
| Dietary health of the child vs affordability | Stressor - Financial stress | "I prefer buying a cheaper brand of jam over a more expensive one. But I do not look at whether one has more sugars or fats than the other." (Participant 8, mother) |
|  | Stressor - Daycare does not meet food wishes | "It is also a healthy daycare. But they also often eat vegetarian or something with fish or egg. I do try to give her a piece of meat on the weekend. That might not be very cheap, but otherwise she [daughter] would not have it at all."(Participant 12, mother) |
|  | Protective factor - Daycare providing most desired foods | "I just think, what is affordable? How can you do it? But of course, you want her to be healthy. And I am lucky that she also gets meals at daycare. So, in the contribution I pay, everything is included—food and diapers." (Participant 12, mother) |
| Dietary health of the child vs well-being of the caregiver | Stressor - Challenging behaviour of the child | "First, I focus on the food. I give them what is healthy, less sugar. [...] If I am tired or sick… They do not listen as strictly anymore. I take a break and calmly talk to them. I say, 'If you listen and behave well for mom, then [you can have or do] whatever you want...' They usually want to go outside or have a treat, like chocolate or something." (Participant 10, mother) |
|  | Stressor - Child rejecting foods | When work is busy or you have still got things on your mind, and then you come home and there is chaos everywhere with toys and everything. And then you think, oh right, I still need to deal with food, and you make something, and suddenly they want something else, and yeah. I just do not have the mental space for it then. [...] No, by then the irritation level is so high that there is just no flexibility left. So then it is just, then you cry. (Participant 13, mother) |
|  | Stressor - No room for me-time | “Participant 3: I used to [eat breakfast with women from the neighbourhood], but now with the kids. Now I have to go back to give them a sandwich. I am busy. So, that is why. Participant 2: Before, I was not so busy. When I did not have a baby, I eat nice breakfast here. It was cozy.” (Participant 2 & Participant 3, mothers) |
|  | Stressor - Change of routine | "Yes, when I am busy sometimes. Yes, just stop whining for a moment. Then you get one of those fruit squeeze pouches. They are not unhealthy in themselves. We should not have ten a day. It is still sugar after all. And when we are outside, then he does take one. He does not hear it, but then he acts up again. Just to keep him quiet for a bit, to keep things sweet. I actually do not want that. But I do it anyway. I notice it more during the holidays. Or when we go outside." (P5, couple , mother) |
|  | Stressor - Treats at daycare & school | "Yes, especially with birthday treats. At school, at after-school care, or at daycare. Then they are allowed to share the treats. If there is a lot of it. […] Yes, because all those candy bags with lots of sweets — if I let them eat all of that, they will be bouncing around all evening." (Participant 15, mother) |
|  | Protective factor - routine | “Routine is really important in life. [...] The kids go to school. You get back to what you are doing. Eat. Make food. Clean up. Pick up the kids. Eat. Have some playtime. Bedtime. [...] Routine with your friend. You can have coffee together. (Participant 3, mother) |
| Dietary health of the child vs social belonging of the caregiver | Stressor - Child rejecting foods | “I secretly think sometimes, like, 'Oh, that child is the same age as mine. They are already eating the same meals as the family, even things like paella with all sorts of ingredients. But mine is not doing that yet.' I have to admit, I found that a bit difficult at first. [...] I think it had more to do with my own insecurities than with it being important for him to eat the same meals as the family” (Participant 8, mother) |
|  | Stressor - Friends & Family providing unhealthy foods | “So yeah, she has M&Ms on the table. [...] But I just let it go because they do not see their grandmas that often. [...] So it is really negligible. If they want to snack on sweets, cakes, and whatever else.” (Participant 16, mother) |
|  | Stressor - Lack of shared health values with other parents | "It is not like you really build friendships there [at community centre meet-ups], or that you get more personal contact. [...] Yeah, you are on your own. And I think maybe also because of the things I’ve been through in life, and because I am older, I do not approach life as casually anymore. [...] They are often young women, who maybe have one, two, or three kids. They handle things in a very carefree way, without much concern. They easily give their kids a cookie or put them in front of YouTube. Yeah, they just handle things very differently." (Participant 12, mother) |
|  | Protective factor - Child eats little of the snack | "Yes, my little sister has kind of spoiled my daughter. Because whenever my sister is around, she says, 'Auntie, shall we go to McDonald's?' Because she knows they always go to McDonald's. But I must say, my daughter is really not an unhealthy eater. Because when she goes to McDonald's, she eats ten fries and two chicken nuggets. And then she is done." (Participant 14, mother) |
|  | Protective factor - Others accept caregiver food rules | "Yes, my parents have positioned themselves in such a way that they go along with what we find important, and there has actually never been any discussion about it. Sometimes, of course, there is a question like, 'Can they really not have a cookie?' or 'Can they really not have that?' but they do accept it.."(Participant 13, mother) |
|  | Protective factor - Role model in social circle | “My sister-in-law has twins, and she is very focused on nutrition and everything related to development, you know. She loves reading and all that. She always said, 'Yeah, you should not give sugar until they are two years old; it is not good.' My mom, on the other hand, was more into giving sugar. For example, honey, which is actually forbidden for children under one year old. But in our culture, we just give it to every child, letting them taste it or something. Or they put chocolate on a pacifier to get the child used to it, you know? She [my sister-in-law] was always against it. And when she had her second child, I was already 15 or 16 years old, so I saw it all happening from her.” (Participant 7, mother) |
